# Repetitive transcranial magnetic stimulation for the treatment of Alzheimer's disease: A systematic review and meta-analysis of randomized controlled trials

**DOI:** 10.1371/journal.pone.0205704

**Published:** 2018-10-12

**Authors:** Xin Dong, Lanyun Yan, Lin Huang, Xinying Guan, Changhong Dong, Huimin Tao, Teng Wang, Xiaoxuan Qin, Qi Wan

**Affiliations:** 1 Department of Neurology, the First Affiliated Hospital of Nanjing Medical University, Nanjing, Jiangsu Province, China; 2 Department of Special Medicine, The First People’s Hospital of Lianyungang, Lianyungang, Jiangsu Province, China; 3 Department of Clinical Medicine, Xuzhou Medical University, Xuzhou, Jiangsu Province, China; Banner Alzheimer's Institute, UNITED STATES

## Abstract

**Background:**

Several studies have demonstrated that repetitive transcranial magnetic stimulation (rTMS) may have a beneficial effect in Alzheimer’s disease (AD). Nevertheless, the clinical benefit of rTMS for AD remains inconclusive.

**Objective:**

This systematic review and meta-analysis aimed to evaluate the efficacy and safety of rTMS in AD.

**Methods:**

We searched PubMed, Embase and Cochrane for randomized controlled trials (RCTs) of rTMS for AD. We calculated pooled estimates of mean difference (MD) with 95% confidence intervals (CI). The protocol was registered at International Prospective Register of Systematic Reviews (PROSPERO) (number CRD42018089990).

**Results:**

Five RCTs involving 148 participants were included in this review. Compared with sham stimulation, high-frequency rTMS led to a significant improvement in cognition as measured by ADAS-cog (MD = -3.65, 95% CI -5.82 to -1.48, p = 0.001), but not MMSE (MD = 0.49, 95% CI -1.45 to 2.42, p = 0.62). High-frequency rTMS also improved the global impression in comparison to the placebo (MD = -0.79, 95% CI -1.24 to -0.34, p = 0.0006). There was no significant difference in mood (MD = -1.36, 95% CI -3.93 to 1.21, p = 0.30) and functional performance (MD = 0.59, 95% CI -1.21 to 2.38, p = 0.52) between high-frequency rTMS and sham groups. Only one trial included low-frequency rTMS reported no significant improvement in cognition, mood and functional performance. Few mild adverse events were observed in both the rTMS and sham groups.

**Conclusions:**

RTMS is relatively well tolerated, with some promise for cognitive improvement and global impression in patients with AD. Our findings also indicate the variability between ADAS-cog and MMSE in evaluating global cognitive impairment.

## Introduction

Alzheimer’s disease (AD) is a devastating neurodegenerative disorder characterized by prominent deficits in memory, language, spatial cognition and executive functioning and changes in mood, personality and behavior. The progressive functional impairment in AD patients imposes a huge burden on healthcare and society [1]. Current pharmacological treatments for AD including the cholinesterase inhibitors and memantine have shown limited efficacy on cognitive function [2–4]. Hence there is an urgent need to identify alternative therapeutic strategies for patients with AD.

Transcranial magnetic stimulation is a neurophysiologic technique for non-invasive stimulation of the brain by inducing electrical current via the principle of electromagnetic induction [5]. An oscillating magnetic field is generated by a strong current circulating within a coil positioned at the skull surface, which produces a corresponding electrical current within the cortical tissue lying under the coil. Repetitive transcranial magnetic stimulation (RTMS) delivers a train of pulses in a rhythmic and repetitive form, which can modulate neural activity and cortical excitability [6]. High-frequency rTMS is considered to stimulate the neuronal activity in targeted cortical regions, while low-frequency rTMS inhibits the neuronal activity of the stimulated areas [7]. RTMS can also have a remote impact on cortical and subcortical structures related to the stimulation site [8]. It has also been observed that rTMS is able to produce after-effects via inducing long-term potentiation or depression on synaptic activity [9]. This technique has therefore been widely investigated as a potential therapy for neurological and psychiatric disorders such as stroke, Parkinson’s disease, epilepsy, depression and schizophrenia [10, 11].

In recent years, several clinical trials have been conducted to investigate the efficacy and safety of rTMS in patients with AD [12–18]. The systematic review focusing on the effects of rTMS in patients with AD has been performed, demonstrating that rTMS may provoke a beneficial effect on cognitive function in AD [19]. Nevertheless, the effects of rTMS on cognition in AD remain inconclusive due to the different study design and outcome measures used across studies. Furthermore, the effects of rTMS on mood, global impression and functional performance that often accompany AD were not investigated in the previous systematic review. Recently two additional randomized controlled trials (RCTs) have been published since the previous systematic review [15, 16]. Therefore, we did a systematic review and meta-analysis of RCTs to evaluate the effects of rTMS on cognition, mood, global impression and functional performance in patients with AD.

## Materials and methods

### Study design and registration

This systematic review and meta-analysis adhered to the Preferred Reporting Items for Systematic Reviews and Meta-analyses (PRISMA) statement (S1 File) [20] and the protocol was registered at International Prospective Register of Systematic Reviews (PROSPERO) (number CRD42018089990).

### Search strategy

We searched the database of PubMed, Embase and Cochrane to find studies published up to May 20, 2018 without language restrictions. We used the following key search terms: “transcranial magnetic stimulation”, “Magnetic Stimulation, Transcranial”, “Alzheimer disease”, “Alzheimer's Disease”, “Alzheimer Dementia” and “Alzheimer's Dementia”. The reference lists of relevant publications were also hand searched. The complete search strategy is listed in S2 File.

### Study selection and data extraction

We regarded studies as eligible for inclusion if they fulfilled the following criteria: (1) Participants were diagnosed as Alzheimer’s disease; (2) RTMS was performed alone or in combination with other treatments; (3) Sham rTMS was applied as a comparison; (4) Outcome measures of cognitive function were quantitatively reported as either the Alzheimer’s Disease Assessment Scale-cognitive subscale (ADAS-cog) or Mini-Mental State Examination (MMSE); (5) Studies were randomized controlled trials.

Two independent investigators (Dong and Yan) screened the titles and abstracts of articles, and studies that potentially satisfied the inclusion criteria were retrieved for full-text assessment. Disagreements were resolved by consensus or by a third reviewer (Huang).

The following information was extracted from the included studies by two independent authors: participant characteristics (total number of participants, age, gender, education, baseline MMSE), rTMS parameters (stimulation targets, frequency, intensity, number of sessions, total pulses per session and sham stimulation), outcome measures, follow-up and adverse effects. The primary outcome measure was cognitive function as measured by ADAS-cog or MMSE. The secondary outcomes included mood as measured by Geriatric Depression Scale (GDS), global impression as measured by Clinician’s Global Impression of Change (CGIC), and functional performance as measured by Instrumental Daily Living Activity (IADL) scale. Baseline data and post-treatment outcome measures were obtained for meta-analysis. In the case of outcomes being reported at multiple time points, the outcome measures immediately after the intervention were used. Where data were missing from the published report, we contacted the corresponding authors of the studies to obtain the data.

### Assessment of risk of bias in included studies

Two reviewers (Dong and Yan) independently assessed risk for bias according to the Cochrane Collaboration’s Risk of Bias Tool [21]. Any disagreements were resolved by discussion or by involving an independent party (Huang). The domains in the Cochrane Collaboration’s tool for assessing the risk of bias include random sequence generation, allocation concealment, blinding of participants and personnel, blinding of outcome assessment, incomplete outcome data, selective outcome reporting and other bias. We classified the risk of bias for each domain as low, high or unclear risk.

### Statistical analysis

We used Review Manager (version 5.3, Cochrane Collaboration) for all statistical analysis. The data arising from ordinal rating scales in the included clinical trials were treated as continuous outcomes and the mean changes from baseline were reported. We estimated the treatment effect using the mean difference (MD) with 95% confidence intervals (CI). If the standard deviations (SD) were not supplied, we computed the missing value based on the p values according to the principles of the Cochrane Handbook for Systematic Reviews of Interventions [22]. Heterogeneity between studies was assessed using Chi^2^ test and I^2^ statistic. For the Chi^2^ test, a significance level was set at P < 0.10. I^2^ values greater than 50% was regarded as significant heterogeneity [23]. If there was no significant heterogeneity (I^2^ < 50%), we calculated pooled estimates of the mean differences between intervention groups by using a fixed-effects model. In the case that heterogeneity was significant, a random-effects model analysis was conducted. Prespecified subgroup analysis was performed based on rTMS stimulation frequency and target sites. Sensitivity analysis was performed with the leave-one-out approach when heterogeneity was significant among studies. Due to the limited number of included studies (less than 10), publication bias was not investigated.

## Results

### Study selection

**Fig 1:**
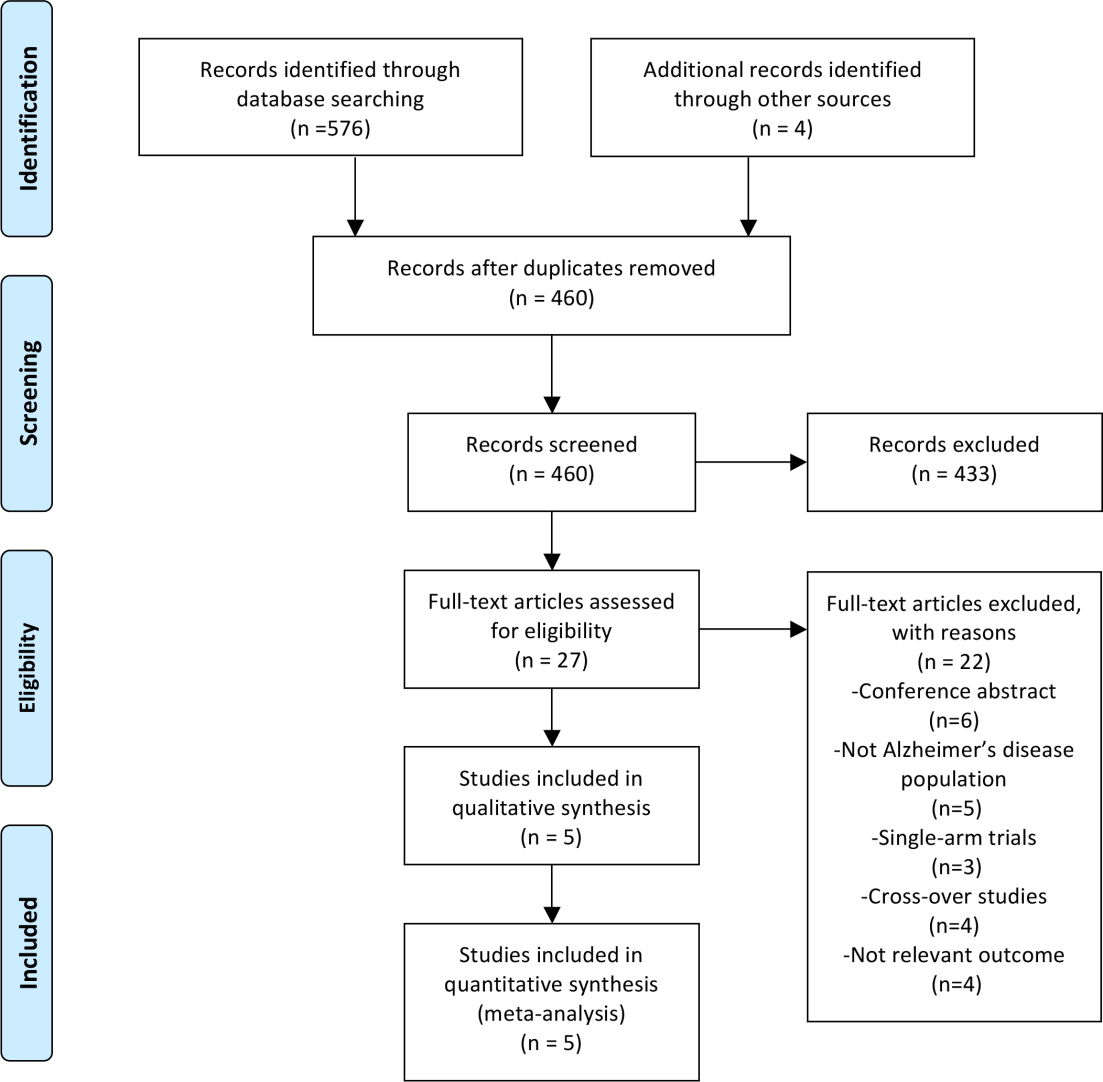
Flowchart for study selection process.

We identified 580 records from the electronic database searches. After removing duplicates, we screened the titles and abstracts of the remaining 460 records and identified 27 full-text articles assessed for eligibility. We included five trials involving 148 participants in the review for quantitative analysis (Fig 1).

### Study characteristics

The characteristics of the included studies are summarized in Table 1. The five trials were all published between 2011 and 2016 [12–16]. Participants were diagnosed with probable AD based on the diagnostic criteria of the Diagnostic and Statistical Manual of Mental Disorders, Fourth Edition (DSM-IV) or the National Institute of Neurological and Communicative Disorders and Stroke Alzheimer’s Disease and Related Disorders Association (NINCDS-ADRDA). Patients had mean baseline MMSE ranging from 13.8 to 22.5. The duration of interventions ranged from 2 weeks to 4.5 months. Three studies assessed the effects of rTMS at different follow-up periods ranging from 1 to 3 months after the last stimulation [12, 13, 16]. Of the studies, only one study included low-intensity rTMS [13] and all other studies used high-frequency rTMS [12–16]. The rTMS was applied to the left dorsolateral prefrontal cortex (DLPFC) [12, 15], bilateral DLPFC [13], or multiple brain regions [14, 16]. All of the trials used a sham stimulation as a placebo control.

**Table 1:** Characteristics of included studies on repetitive transcranial magnetic stimulation for Alzheimer’s disease in the meta-analysis.

Study	Number of participants (stim/sham)	Gender (male/female)	Mean age (years)	Mean education (years)	Mean baseline MMSE	Stimulation target	Stimulation protocol	Sham stimulation	Outcome measures	Follow-up	Adverse effects
Cotelli 2011	5/5	Not available	72.8	5.6	16.1	L DLPFC	20Hz, 100% RMT, 2000 pulses per session, 10 sessions over 2 weeks	Sham coil	MMSE IADL	8 weeks	No
Ahmed 2012	30/15	16/29	67.6	Not available	13.8	Bilateral DLPFC	(1) 20 Hz, 90% RMT, 2000 pulses per session, 5 daily sessions (2) 1 Hz, 100% RMT, 2000 pulses per session, 5 daily sessions	Coil away from the head	MMSE GDS IADL	1 and 3 months	No
Rabey 2013	7/8	10/5	74.1	Not available	22.0	Broca, Wernicke, L/R DLPFC, L/R pSAC	10 Hz, 90–110% RMT, 400pulses for 2 brain sites and 500 pulses for 1 brain site per session, 5 sessions per week for 6 weeks and 2 sessions per week for 3 months	Sham coil	ADAS-Cog CGIC	No	No
Wu 2015	26/26	21/31	71.7	11.5	15.3	L DLPFC	20Hz, 80% RMT, 1200 pulses per session, 20 sessions over 4 weeks	Coils turned 180 degrees	ADAS-Cog	No	Mild extrapyramidal reactions, headache
Lee 2016	18/8	15/11	71.6	9.9	22.5	Broca, Wernicke, L/R DLPFC, L/R pSAC	10 Hz, 90–110% RMT, 400pulses for 3 brain sites per session, 30 sessions over 6 weeks	Sham coil	ADAS-Cog MMSE GDS CGIC	6 weeks	Mild headache, fatigability

stim, stimulation group; sham, sham group; R, right; L, left; DLPFC, dorsolateral prefrontal cortex; pSAC, parietal somatosensory association cortex; RMT, resting motor threshold; MMSE, Mini-Mental State Examination; ADAS-Cog, Alzhermer Disease Assessment Scale-cognitive subscale; IADL, Instrumental Daily Living Activity; GDS, Geriatric Depression Scale; CGIC, Clinician’s Global Impression of Change

### Risk of bias in included studies

**Fig 2:**
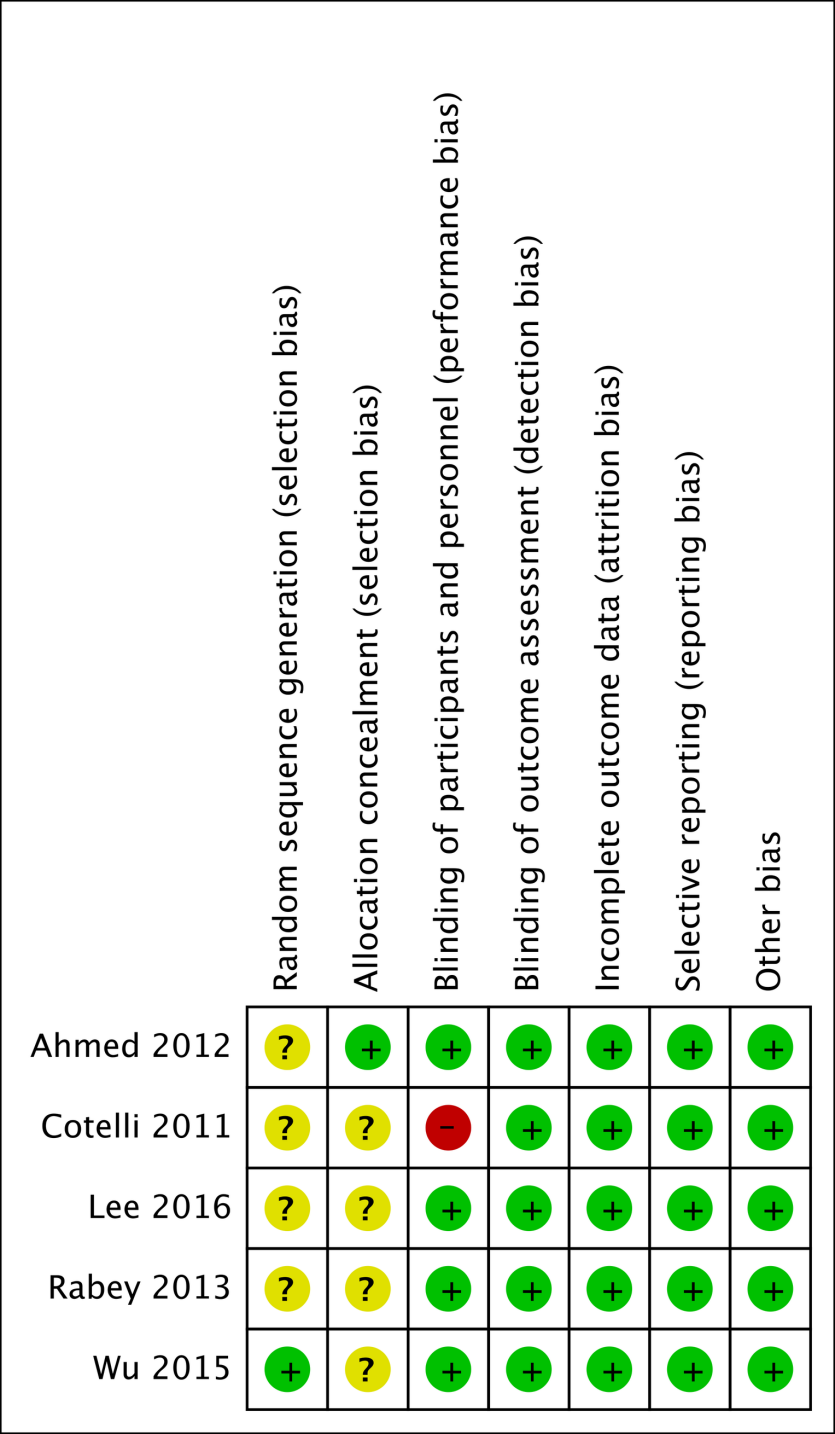
Risk of bias summary of included studies.

The risk of bias of the included studies was summarized in Fig 2. One trial described the method of random sequences generation [15] while other four trials stated randomization to be performed without description of the method [12–14, 16]. Only one study adequately reported allocation concealment [13]. Participants and outcome assessors are blind to treatment allocation in most of the studies except for Cotelli 2011 [12]. The studies of Rabey 2013 and Lee 2016 provided reasons for missing outcomes, and dropout rates were comparable between the treatment and control groups [14, 16]. Therefore, we considered all trials to be at low risk of attrition bias due to incomplete outcome data. All studies reported low risk of reporting bias and other potential sources of bias.

### Primary outcomes

#### Cognition

**Fig 3:**
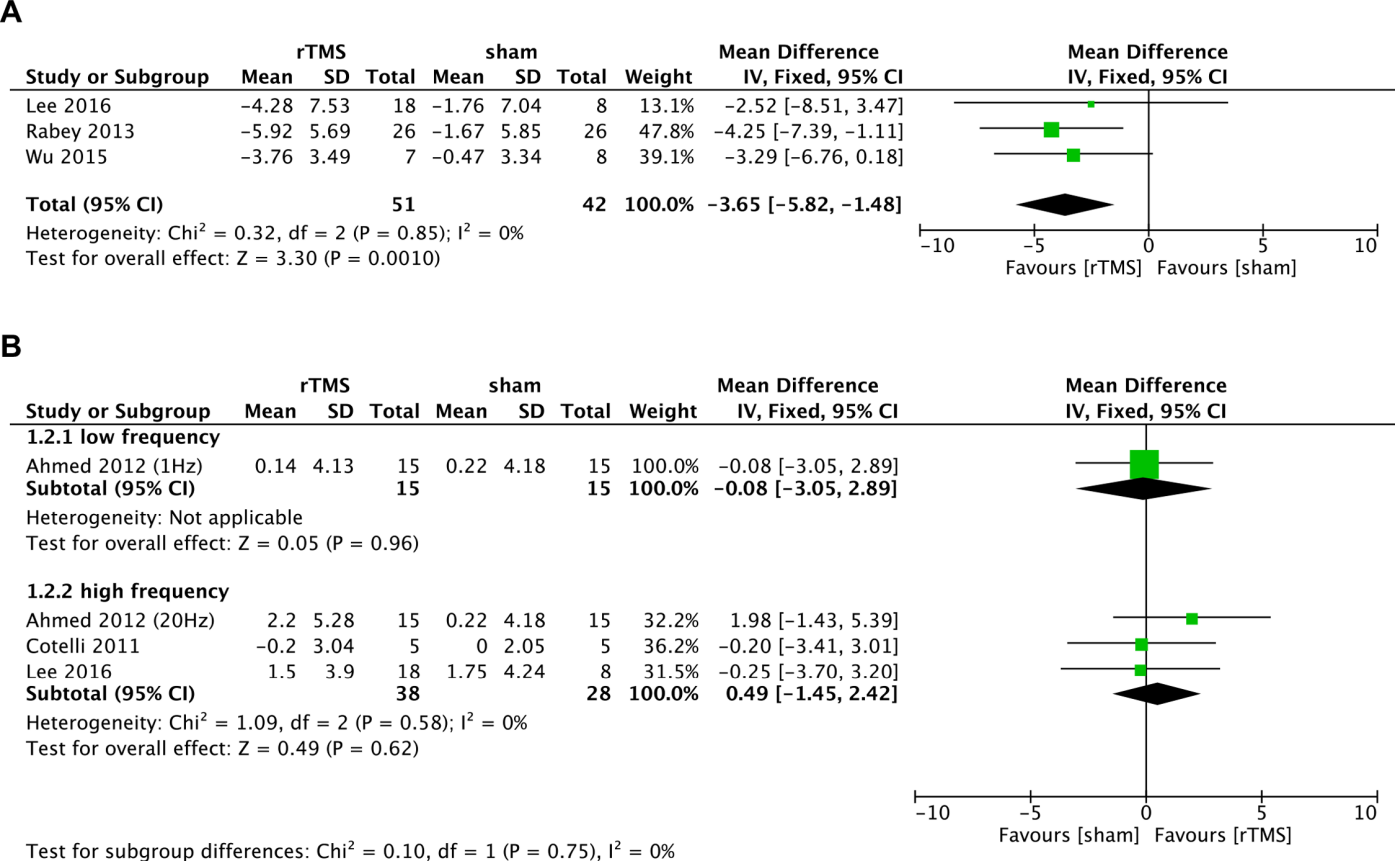
Meta-analysis of cognition after repetitive transcranial magnetic stimulation (rTMS) versus sham rTMS. (A) ADAS-cog. (B) MMSE.

All five studies involving 148 participants with AD assessed the change of cognitive function after treatment as measured by the ADAS-Cog or MMSE. Among these studies, three assessed the effect of rTMS on cognitive function using ADAS-Cog scores [14–16]. Pooling the data of these studies showed that high-frequency rTMS treatment led to a significant improvement in cognitive function as assessed by the ADAS-Cog scores compared with placebo (MD = -3.65, 95% CI -5.82 to -1.48, p = 0.001), with no between-study heterogeneity (I^2^ = 0%) (Fig 3A).

Three studies reported data on the effect of rTMS on MMSE [12, 13, 16]. Subgroup analysis showed no significant improvement in MMSE scores in the high-frequency rTMS group compared with sham stimulation (MD = 0.49, 95% CI -1.45 to 2.42, p = 0.62, I^2^ = 0%) (Fig 3B). Only one trial [13] included low-frequency rTMS showed no statistically significant effect on MMSE (MD = -0.08, 95% CI -3.05 to 2.89, p = 0.96) (Fig 3B).

### Secondary outcomes

#### Mood

**Fig 4:**
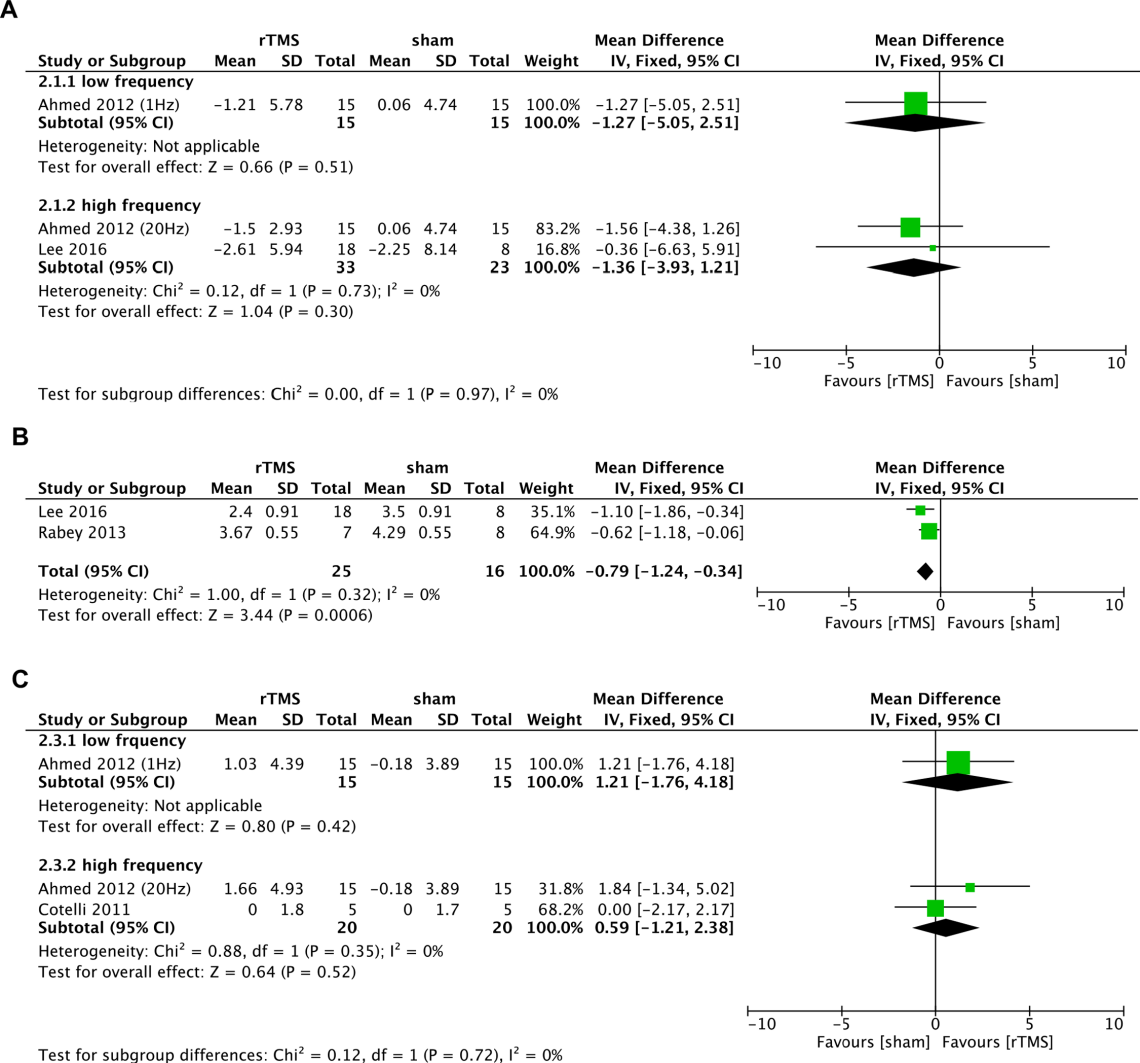
Meta-analysis of mood, global impression, function after repetitive transcranial magnetic stimulation (rTMS) versus sham rTMS. (A) Change in mood as measured by GDS. (B) Change in global impression as measured by CGIC. (C) Change in functional performance as measured by IADL.

Only two studies that included a total of 71 participants provided data on mood, and all assessed depression as a mood symptom using GDS scale [13, 16]. Pooled analysis of the data in the two studies did not show any benefit of high-frequency rTMS for mood compared with sham stimulation (MD = -1.36, 95% CI -3.93 to 1.21, p = 0.30). We detected no between-study heterogeneity (I^2^ = 0%) (Fig 4A). Only one trial [13] included low-frequency rTMS reported no significant improvement in mood (MD = -1.27, 95% CI -5.05 to 2.51, p = 0.51) (Fig 4A).

#### Global impression

Two studies assessed global impression using the CGIC scale [14, 16]. There was a significant difference between high-frequency rTMS and sham groups in favor of rTMS for global impression immediately after the treatment (MD = -0.79, 95% CI -1.24 to -0.34, p = 0.0006), with no heterogeneity between studies (I^2^ = 0%) (Fig 4B).

#### Functional performance

The meta-analysis of two trials that assessed the functional performance by IADL scale [12, 13] showed no statistically significant difference in functional performance between high-frequency rTMS and sham groups immediately after the intervention (MD = 0.59, 95% CI -1.21 to 2.38, p = 0.52), with no significant heterogeneity (I^2^ = 0%) (Fig 4C). There was no significant effect of low-frequency rTMS on functional performance (MD = 1.21, 95% CI -1.76 to 4.18, p = 0.42) (Fig 4C).

### Adverse effects

Two studies reported adverse effects related to the application of rTMS [15, 16]. Wu et al. reported four cases of mild extrapyramidal reactions and four cases of transient headache in the rTMS intervention group, with no significant difference compared with the sham group [15]. Lee et al. described one patient who experienced mild headache and fatigability in the sham group [16]. The remaining three studies did not report any adverse effects [12–14].

## Discussion

The systematic review shows that high-frequency rTMS can significantly improve the cognition as measured by ADAS-cog, but not MMSE. High-frequency rTMS also improves the global impression in comparison to the placebo, whereas, rTMS does not show any benefit for mood and functional performance in patients with AD. In addition, rTMS is safe and well tolerated compared with sham stimulation. The results also indicate the difference between instruments of ADAS-cog and MMSE for assessing cognitive function in these patients.

In this meta-analysis, five RCTs including 148 participants comparing the efficacy of rTMS with control group revealed the therapeutic benefits of rTMS in patients with AD. Overall, we were able to obtain and extract the primary outcomes of cognitive function from all the included trials. However, not all the included studies reported data on our pre-determined secondary outcomes, such as mood, global impression, and quality of life. Thus, additional research is required to explore the effects of rTMS on other clinically relevant outcomes in AD. In addition, the parameters including stimulation frequency, location and duration used for the rTMS in these RCTs were different. It does not allow for selection of optimal parameters to improve the cognitive function in AD. The stimulation frequency of rTMS has a huge influence on the efficacy of rTMS in Alzheimer's disease. High-frequency rTMS can produce after-effects via inducing long-term potentiation (LTP) on synaptic activity [9], which is considered to be a critical central cellular mechanism of learning and memory [24]. In the present study, pooled analysis of the five RCTs that investigated the efficacy of high-frequency rTMS for AD showed that high-frequency rTMS was well tolerated and effective on cognitive function as measured by the ADAS-Cog [12–16]. Only one trial included low-frequency rTMS for the treatment of AD, which reported that no statistically significant effect was found between low-frequency rTMS and sham stimulation [13]. Thus, we considered that high-frequency stimulation may reach an obvious effect. However, these results should be interpreted with caution owning to the relatively small number of trials, particularly for low-frequency rTMS. Besides, various stimulation targets across studies also contributed to the variability. The most common stimulation target of rTMS for cognitive function in patients with AD was DLPFC [12, 13, 15]. Moreover, two studies reported that stimulation of six functional regions of the brain also revealed a benefit for cognitive function [14, 16]. It has been widely known that the prefrontal cortex plays a critical role in cognitive functions [25], which is abnormally disturbed in AD [26]. Therefore, the use of rTMS over DLPFC may improves cognition by a direct stimulation of this cortical area and activation of connected circuits in remote structures. These six brain regions represent the primary cortical locations that are closely related with the clinical manifestation of AD, including the bilateral DLPFC, Broca’s and Wernicke’s areas, and bilateral parietal somatosensory association cortex (pSAC). A recent study found that rTMS over the left DLPFC and the six brain regions was equally effective on cognitive function [27]. Further data are required to optimize the target sites of rTMS for AD. With regard to the effects of rTMS for AD, the long-term effects also need to be investigated in addition to immediate effect. All included studies assessing the changes of cognition immediately after the intervention suggested a possible short-term effect of rTMS for AD. Only two studies measured the cognitive outcomes at the follow-up period ranging from 1 month to 3 months after stimulation [13, 16]. Further well-designed RCTs should be performed to optimize the stimulation parameters to evaluate the short-term and long-term efficacy of rTMS for AD.

Only few data are available reporting possible clinical effects of rTMS in patients with AD. After an extensive search, we identified one meta-analysis on rTMS treatment for AD [19]. The systematic review by Liao et al. addressed the efficacy of rTMS on cognitive function in patients with AD, which was in agreement with the cognitive improvement on the ADAS-cog in our meta-analysis [19]. Nevertheless, rTMS did not show similar cognitive benefit for the MMSE in our present meta-analysis. The substantial difference between MMSE and ADAS-cog data may be subjected to the dementia severity of patients among studies and the intrinsic variance of the instruments used, comprising various subdomains of cognition. MMSE is a brief screening tool assessing global cognitive impairment, and ADAS-cog score is more precise in exploring the cognitive function than the MMSE scale [28]. The different instruments used across studies limits the ability to calculate the pooled effect size among studies. In addition to cognition, our present meta-analysis also investigated the effects of rTMS on mood, global impression and functional performance in AD patients. Moreover, two recent published RCTs have been included in our meta-analysis since the previous review [15, 16]. The previous systematic review included both cross-over studies and self-controlled studies while our present review only included trials with parallel design [29, 30]. AD is a degenerative disorder that patients can deteriorate rapidly over time during the trial [31], and rTMS can have a lasting effect that may persist into a subsequent period [32]. Thus, only data from the first period of the cross-over studies can be extracted for analysis to minimize the risk of carry-over [33]. In the present systematic review, cross-over trials were excluded for meta-analysis for it is impossible to extract outcome data from the first treatment period.

A limitation of our meta-analysis is that the sample size was too small to ensure adequate power to detect a significant difference in primary outcomes among groups [34]. The included trials ranged in sample size from 15 to 52 participants. Secondly, the rTMS stimulation parameters varied across the included trials. We cannot propose a consistent protocol in relation to stimulation frequency, targets and duration. Thirdly, the measurement scales for cognitive function across the studies were inconsistent, and other clinical outcomes related to mood, global impression and functional performance were insufficient to draw a definite conclusion. Additionally, most studies did not provide clear information on study design, particularly the methods of randomization and allocation concealment, with an unclear risk of bias. Only one study reported the method of randomization [15] and one pointed out the method of allocation concealment separately [13]. The quality of evidence for outcomes may be rated as low due to the unclear risk of bias in the included studies. Further research is very likely to have an important impact on our confidence in the estimate of effect and is likely to change the estimate. Further high-quality studies with low risk of bias are needed to better discuss the efficacy and safety of rTMS in AD. Finally, subgroup analysis based on rTMS stimulation target sites was not performed in the meta-analysis due to the limited number of included trials.

In conclusion, rTMS is relatively well tolerated, with some promise for cognitive improvements and global impression in patients with AD. The results also indicate the variance between ADAS-cog and MMSE score in evaluating global cognitive function. Nevertheless, it is not possible for us to draw a definite conclusion due to the limitations of included studies. Methods of randomization and allocation concealment should be clearly provided in future trials to investigate the efficacy of rTMS as a sole or adjuvant therapeutic strategy in patients with AD. Further studies with large sample sizes and long-term follow-up are needed to establish the optimal protocol and to assess the long-term efficacy of rTMS in AD.

## Supporting information

S1 File PRISMA 2009 checklist. (DOC)

S2 File Search strategies. (DOC)
