# Supplementary material for: Repetitive transcranial magnetic stimulation for the treatment of Alzheimer's disease: A systematic review and meta-analysis of randomized controlled trials
Source: PLoS One. 2018 Oct 12;13(10):e0205704. doi: 10.1371/journal.pone.0205704 (PMC6185837; doi:10.1371/journal.pone.0205704)
Supplement: S2 File — (DOC) [file pone.0205704.s002.doc]

**Search strategy for Pubmed**

#1 "Alzheimer Disease"[Mesh]

#2 (((Alzheimer Disease[Title/Abstract]) OR Alzheimer's Disease[Title/Abstract]) OR Alzheimer Dementia[Title/Abstract]) OR Alzheimer's Dementia[Title/Abstract]

#3 #1 OR #2

#4 "Transcranial Magnetic Stimulation"[Mesh]

#5 (Transcranial Magnetic Stimulation[Title/Abstract]) OR Magnetic Stimulation, Transcranial[Title/Abstract]

#6 #4 OR #5

#7 "randomized controlled trial"[pt] OR "controlled clinical trial"[pt] OR randomized[tiab] OR placebo[tiab] OR "drug therapy"[sh] OR randomly[tiab] OR trial[tiab] OR groups[tiab]

#8 #3 AND #6 AND #7

**Search strategy for Embase**

#1 'alzheimer disease'/exp

#2 'dementia'/exp

#3 'alzheimer*':ab,ti OR 'dement*':ab,ti

#4 #1 OR #2 OR #3

#5 'transcranial magnetic stimulation'/exp

#6 'transcranial magnetic stimulation*':ab,ti OR 'tms':ab,ti OR 'rtms':ab,ti

#7 #5 OR #6

#8 'randomized controlled trial'/exp OR 'controlled clinical trial'/exp OR 'randomized':ti,ab OR 'placebo':ti,ab OR 'drug therapy':lnk OR 'randomly':ti,ab OR 'trial':ti,ab OR 'groups':ti,ab

#9 #4 AND #7 AND #8

**Search strategy for Cochrane Library**

#1 MeSH descriptor: [Alzheimer Disease] explode all trees

#2 MeSH descriptor: [Dementia] explode all trees

#3 (alzheimer* or dement*):ti,ab,kw (Word variations have been searched)

#4 #1 or #2 or #3

#5 MeSH descriptor: [Transcranial magnetic stimulation] explode all trees

#6 Transcranial magnetic stimulation:ti,ab,kw (Word variations have been searched)

#7 #5 or #6

#8 #4 and #7
